# Persisting stigma reduces the utilisation of HIV-related care and support services in Viet Nam

**DOI:** 10.1186/1472-6963-12-428

**Published:** 2012-11-25

**Authors:** Duong Cong Thanh, Karen Marie Moland, Knut Fylkesnes

**Affiliations:** 1 National Institute of Hygiene and Epidemiology, Ha noi, Viet Nam; 2 Centre for International Health, University of Bergen, Bergen, Norway; 3 Faculty of Health and Social Sciences, Bergen University College, Bergen, Norway

## Abstract

**Background:**

Seeking and utilisation of HIV prevention, treatment, care, and support services for people living with HIV is often hampered by HIV-related stigma. The study aimed to explore the perceptions and experiences regarding treatment, care, and support amongst people living with HIV in Viet Nam, where the HIV epidemic is concentrated among injecting drug users, sex workers, and men who have sex with men.

**Methods:**

In-depth interviews and focus group discussions were conducted during September 2007 in 6 districts in Hai Phong with a very high HIV prevalence among injecting drug users. The information obtained was analysed and merged within topic areas. Illustrative quotes were selected.

**Results:**

Stigma and discrimination against people living with HIV in the community and healthcare settings was commonly reported, and substantially hampered the seeking and the utilisation of HIV-related services. The informants related the high level of stigma to the way the national HIV preventive campaigns played on fear, by employing a “scare tactic” mainly focusing on drug users and sex workers, who were defined as “social evils” in the anti-drug and anti-prostitution policy. There was a strong exclusion effect caused by the stigma, with serious implications, such as loss of job opportunities and isolation. The support and care provided by family members was experienced as vital for the spirit and hope for the future among people living with HIV.

**Conclusions:**

A comprehensive care and support programme is needed. The very high levels of stigma experienced seem largely to have been created by an HIV preventive scare tactic closely linked to the “social evil“ approach in the national policy on drug and prostitution. In order to reduce the stigma and create more effective interventions, this tactic will have to be replaced with approaches that create better legal and policy environments for drug users and sex workers.

## Background

Care and support for people living with HIV (PLHIV) is an issue of great importance for quality of life, treatment success and HIV prevention. Evidence suggests that access to care and support is associated with psychological well-being [1-3], reduced HIV risk behaviour [4-6], HIV status disclosure [7,8], use of health services [6,9], and antiretroviral treatment (ARV) outcomes [10] among PLHIV. Family support has a positive effect on medical and treatment decisions, and with family relations, hope, and the person’s attitude towards life in general [11]. There is a wide range of family support projects for PLHIV including financial assistance, support in the disclosure process, daily routine activities, medical assistance, and psychological support [11]. Social support can be divided into emotional support which nurtures a sense of belonging and personal worth; informational support which increases awareness and knowledge; and instrumental support, which is practical assistance with daily living [12].

Stigma is a significant barrier to HIV prevention, treatment, care and support for PLHIV, and efforts to reduce it seem limited [13]. Stigmatisation, as a ‘process of devaluation’ of people either living with or associated with HIV/AIDS, is expressed through discrimination of various forms and at different levels including family, community, institutional, and national levels [14]. Stigma can be perceived or felt, and it can be enacted. Where stigma refers to actual discrimination it is enacted, stigma referring to the fear of such discrimination is felt [15]. HIV-related stigma and discrimination may occur on family, community, institutional, and national levels, and may be experienced as social isolation, physical violence, verbal harassment and political discrimination [14,16]. Stigma is associated with drug use, decreased HIV disclosure to sex partners, social network members that may decrease condom use in HIV discordant couples, and non-adherence to HIV treatment [17]. HIV-related stigma presents an obstacle to getting tested for HIV, obtaining optimal HIV care, and engaging in safe sex practices [18]. HIV-related stigma and discrimination are believed to be widespread in Viet Nam [19,20].

The HIV epidemic in Viet Nam is concentrated primarily among injecting drug users (IDUs), female sex workers (FSWs), and men who have sex with men (MSM) [21,22]. From the early years of the HIV epidemic, the government has promoted a counselling and care programme; the QTC (Quản lý-Tư vấn-Chăm sóc or Management-Counselling-Care) [23], which aims to provide post-test counselling and care services to people tested HIV-positive. A comprehensive home visiting programme has been instituted. Register information about HIV-positive individuals is disseminated through the vertical healthcare system, from the provincial level to the district and then to the communal health centre. A local healthcare worker in charge of the HIV programme at a communal health centre will then visit each person at home to provide counselling and referral services. Mass organizations such as the Viet Nam Women’s Union (VWU), the Viet Nam Youth Union (VYU), and the Viet Nam Red Cross (VRC) are other important agencies providing care, support and home visits to PLHIV. So far, the government has not offered any training programmes or financial support to family or community caregivers. There are examples, however, of international non-governmental organizations (NGOs) having offered financial and technical support for implementing services [23]. A National Action Plan on HIV/AIDS Care and Treatment was approved in 2006 and remains operational [24]. The government’s commitment to increasing access to treatment is reflected in a substantial increase in the annual domestic budget for ARV. Despite this significant increase, donors still constitute a major source of funding for treatment [23].

The present study was conducted in Hai Phong city, located in the economic developmental triangle of Quang Ninh-Hai Phong-Ha Noi. This triangle was the centre of the HIV epidemic among IDUs in the North. The prevalence of HIV among IDUs in this area exploded in the late 1990s and seems to have peaked around 2001–2002, with 75% of IDUs being HIV-positive in Quang Ninh in 2002, 72% in Hai Phong in 2001 and 31% in Ha Noi in 2004. Later observations indicated a decreasing trend, but HIV prevalence among IDUs in these provinces remains very high. The HIV prevalence among antenatal women in Quang Ninh has exceeded 1% [25].

In response to the serious HIV epidemic in the area, the local authorities in Hai Phong with support from the central government and various international donors have developed intervention programmes e.g. peer education and self-help groups, and care and treatment programmes. The peer education programmes are based on outreach activities that include individual counselling, condom promotion, distribution of needles and syringes and distribution of educational pamphlets and brochures. The self-help groups aim to reduce stigma and discrimination, and improve the quality of life of PLHIV, as well as to raise their self-esteem. The care and treatment programmes provide anti-retroviral therapy, treatment for opportunistic infections (OIs), and other related services, including medical provider training and capacity building for PLHIV. Despite these staunch efforts, under utilisation of services remains a big problem among PLHIV [26].

Drug use and sex work is illegal in Viet Nam. Because HIV transmission is concentrated in the two groups of IDUs and FSW , PLHIV are often described and treated as “social evils” by the society at large resulting in strong scare tactics in prevention [27,28]. However, it is likely that preventive campaigns that play on fear may be promoting counter-productive behaviour that hampers HIV prevention and utilisation of essential services. This paper aims to explore how PLHIV perceive and experience stigma and discrimination and how this affects their health seeking behaviour and utilisation of HIV treatment, care and support.

## Methods

In order to look for diversity in perspectives and experiences related to stigma, health seeking and utilisation of HIV prevention, treatment, care and support among PLHIV, a qualitative design was deemed appropriate. In-depth interviews and focus group discussions were undertaken among PLHIV to explore individual and group experiences and perceptions.

### Study population and sampling methods

The study was conducted in Hai Phong in the North of Viet Nam between August and September 2007. The city is known to harbour a large number of IDU and PLHIV. The participants were selected from 3 urban districts (Hong bang, Ngo quyen, and Le chan) – those with a relatively high HIV prevalence among IDUs [29] - and from 3 suburban districts (An duong, An lao and Thuy nguyen) selected randomly among the remaining districts in Hai Phong.

In Vietnam, all HIV reactive samples are sent to either the provincial HIV confirmatory laboratories or the regional reference laboratories for confirmation. This data forms the basis for identifying and registering all HIV-positive persons. The test results were to be kept confidential and shared only with participating healthcare professionals and the HIV infected person. Local health workers and peer educators among PLHIV identified through local health workers and PLHIV self-help groups were engaged in recruiting participants. A local health worker and a peer educator in each district were used as ‘seeds’. Participants were first approached and selected based on the registry by the seeds. Some participants were on the list but the team did not have the relationship to make contact and approach them. In these cases “snowball” referrals were employed to recruit the participants. To be included in the study, participants had to be over 15 years old, HIV-positive and live in the study area. To ensure a wide range of information, efforts were made to balance the numbers by their gender, age and education levels.

### Data collection

An interview guide for the in-depth interviews and a topic guide for the focus group discussions were developed. The main points discussed in both the in-depth interviews and focus groups were: the perception of the causes of stigma, the expressions and forms of stigma, and the effects of stigma and discrimination on PLHIV. All focus group discussions and interviews were conducted in Vietnamese. The first author conducted 15 in-depth interviews. All interviews focused on individual perceptions and experiences, and were conducted in private places that facilitated dialogue and were not too noisy for tape-recording. Three focus group discussions were conducted at a private location convenient for the participants. There were 10 participants in each group. The peer educators and the first author acted as moderators, facilitated the discussion and kept it focused on issues related to stigma and discrimination. The focus group discussions were organised separately for women and men. The discussions revolved around the norms, perceptions and experiences of stigma, and were used to pursue issues that had been raised in the in-depth interviews. All in-depth interviews and focus group discussions lasted about 1.5 hours. They were tape-recorded and transcribed immediately after each session by the first author.

Forty-five study participants, 33 males and 12 females between 18–46 years of age, were included in the study. Their educational level was relatively high; one had completed college, 32 had completed secondary school or higher, and 12 had only completed primary school or less. Eleven were unemployed, 20 were married, and eight reported to be under ARV. Twenty-seven were IDUs and the others were non-IDU men and women.

### Data management and analysis

To get familiar with the data, the first author read through the transcripts of the in-depth interviews and the focus group discussions several times. The interviews and FGDs were broken down into meaning units and coded. The codes were grouped in broad categories that were partly pre-defined and partly constructed on the basis of the data. These included: 1) perception of causes of stigma, 2) the expression and forms of stigma, 3) the effects of stigma and discrimination on health seeking behaviour, 4) the effect of stigma and discrimination on self-esteem, 5) the experience of care and support in the healthcare system, 6) the experience of care and support in the local community, and 7) the experience of care and support from family. A summary was made for each category, building on the common and recurrent responses as well as on conflicting viewpoints. The work was translated into English. The categories with summaries and coded meaning units were carefully reviewed by two authors. Three themes were identified, and illustrative quotations were selected. The themes identified included: 1) the public discourse on stigma and discrimination, 2) experiences of seeking and utilisation of healthcare services, and 3) experiences of interaction and support in everyday life. No computer programme was used in the analysis.

### Ethical issues

The study was conducted with the understanding and consent of each participant, and was approved by the Institutional Review Board of the National Institute of Hygiene and Epidemiology, Hanoi. Informants were informed beforehand and consented to the use of tape recorder during interviews and discussion. They were informed about their right to withdraw at any time during the research and that the findings would be kept confidential. Informants volunteered to participate in the focus group discussions without the fear of HIV status disclosure. No personal identifiers were collected.

## Results

The following section explores how PLHIV talked about and made sense of the experiences of care and support as family and community members, and as HIV-positive citizens in contemporary Viet Nam. Their experiences tended to be framed in a language of stigma and discrimination on the one hand, and on care and support on the other. The representations of their experiences of support versus discrimination seemed to vary with relational and emotional distance.

### Public discourse on ‘stigma and discrimination’

At the core of the PLHIV’s narratives of their experiences living with HIV was the concept of ‘stigma and discrimination’. All informants interpreted their experience in terms of this concept either as a lived experience (enacted) or as an expectation (felt). Many expressed that a collective fear of HIV permeated all social interaction and that the fear of acquiring the virus through everyday contact with infected people was a major obstacle to their living a normal life.

#### Negative images

Fear of the disease was stimulated and reinforced by the negative images used by the media, and in health campaigns and health education posters. With the aim of scaring people from engaging in risky behaviour, the information disseminated through the government system tended to nurture negative images of PLHIV. The campaigns using posters with skeletons and messages like ‘stay out of drug and prostitution – the routes to AIDS’, tended to place the responsibility and the blame on IDUs and FSWs for being the source of the virus and causing its spread. Many of the PLHIV thought of this approach as a major cause of stigma and discrimination because, as one informant commented: *“People get scared when they look at them [the images], (a 28-year-old woman, focus group discussion).* Despite the steadily increasing access to ARV as a life-extending drug, the idea that *“HIV/AIDS is not a curable disease, [it] is a death sentence”* voiced by a 38-year-old man in a focus group discussion, was widespread. The concept of a “death sentence” came out as important in the local discourse on HIV, and with its reference to punishment and death it nurtured the fear of the virus and those who were suspected to be infected. These were primarily drug users and sex workers.

#### ‘Social evils’

The strong link made between PLHIV and practices that are considered improper and immoral in mainstream Vietnamese society, like drug abuse and commercial sex, was seen to further sever the ties between the community at large and the individual PLHIV. In the anti-drug and anti-prostitution policy climate in Viet Nam, IDUs and FSWs were coined as ‘social evils’; their behaviour was portrayed as abnormal, and a threat to the safety and health of the general population. The long standing ‘social evil’-status of the IDUs and the FSWs hence was inevitably spread to PLHIV and the 2 marginal statuses came to reinforce one another.

As one of the male participants explained about the views of the population at large:

“Among PLHIV, women are often sex workers and have multiple sex partners, men are often drug users and have multiple sex partners. They are abnormal people.” (A 46-year-old man, focus group discussion)

But the marginal status of the IDUs and FSWs transferred to PLHIV was not only connected to ‘abnormal sexual practices’. In line with the prejudiced images of IDUs and FSW, PLHIV were also associated with bodily and moral decay; with dirt, dishonesty and ultimately with crime:

“In the general public opinion, HIV infection is due to drug addiction. Drug users often steal things. They are often homeless and look dirty. Thus, PLHIV are stigmatised.” (A 27-year-old man, in-depth interview)

The close connection between drug addiction and HIV nurtured a social evil approach to the problem. The basic idea of HIV as a disease of morally marginal people was reflected in the experience of unmet needs and in the interaction with healthcare workers, community members and work-mates.

### Experiences of seeking and utilisation of healthcare services

#### Perceived neglect

The perceived unmet needs for healthcare were partly explained in terms of stigma and discrimination by healthcare workers. Many reported experiences interpreted as expressions of HIV-related stigma and discrimination, such as unfriendly attitudes and neglect, as is illustrated in the quote below:

“Two months ago, I got fever and headache. I went to the district hospital. Healthcare workers knew that I am HIV-positive. They said that they did not treat patients like me. They did not ask me anything. Their attitude toward me was unfriendly. They did not make any examination. They gave me some medicines and asked me to go to the Tuberculosis hospital.” (A 22-year-old man, in-depth interview)

This was seen as a common problem, not only causing suffering on the part of PLHIV, but also affecting their motivation to seek healthcare services.

#### Fear and distrust

Some informants were very critical about the organisation of the HIV related healthcare services. In particular, assigning a separate room for PLHIV, as was common in the hospitals, was interpreted as discrimination. The seclusion of PLHIV achieved in this manner was experienced as exclusion, and was perceived as a way to protect the general public, the ‘normal’ population from exposure to ‘non-normal’ PLHIV. The frustration of being treated as different by other patients is indicated in the following quote:

“I was sick and went to hospital. They knew that I am HIV-positive and I was moved to a separate room.” (A 18-year-old woman, in-depth interview)

Many informants reported that they avoided using healthcare services in fear of being stigmatised and discriminated against:

“My company has a regular health check up programme for employees. I always lie that my kid is sick when we have a check up schedule.” (A 26-year-old woman, in-depth interview)

The trust in health personnel keeping confidentiality was limited in many respondents:

“I am reluctant to go to healthcare services because it may leak my HIV-positive status. This information leaking may affect my job and my children at school.” (A 36-year-old man, in-depth interview)

Fear of HIV status disclosure in healthcare settings was seen to further hamper healthcare seeking and utilisation among PLHIV.

### Experiences of interaction and support in everyday life

#### Exclusion at the workplace

Many PLHIV were concerned about limited job opportunities or the possibility that a disclosed HIV-positive status might lose them their jobs.

“I hide my HIV-positive status to non-family members. If people in my workplace know that I am HIV-positive perhaps I will have to stay at home. I can not stand the eyes of people looking at me like that. Maybe I will lose my job.” (A 25-year-old woman, in-depth interview)

Many informants felt they were squeezed out from work because their work mates feared being infected:

“I want to go back to my previous job as a construction worker. But people at my workplace said that they fear getting HIV infection through an accident. I feel they did not want me to work here for that reason. They then told me that I should stay home because of my health status.” (A 28-year-old man, in-depth interview)

There were also reports of verbal abuse, demonstrations of contempt and attempts at exclusion. Some work mates tended to exert pressure on the PLHIV to stay off work, indicating that work would be wasted on them since they would anyway soon die. One of the informants recalled the following utterance of a work-mate:

”You are about to die; why do you need to work?” (A 40-year-old man, in-depth interview)

The double status as HIV-positive and a drug addict was particularly discouraging in terms of seeking a job:

“I am both HIV-positive and a drug addict. Therefore, I do not dare to seek a job.” (A 32-year-old man, in-depth interview)

Non-disclosure to non-family members was seen as important to prevent unwanted consequences such losing the job. PLHIV who were self-employed feared losing customers and hence ruining their business:

“I had a food-stand before, but people did not come to buy food at my food-stand. I had to close then.” (A 28-year-old woman, in-depth interview)

Although many informants expected discrimination from their bosses at work and feared being sacked, few reported such a negative experience. On the contrary, some reported having been received with understanding and support:

“My HIV status is known only by the board of directors. They asked me if I have any demand or need such as working in another position that is suitable for my health status. I even receive special gifts for those with difficulties on some special occasions.” (A 36-year-old man, in-depth interview)

#### Fear of contagion and isolation in the community

In some cases neighbours were terrified of contagion and normal interaction became impossible. One informant said that neighbours feared being infected also by the dead body of an HIV-positive person:

“When my husband died, neighbours did not allow us to bury him as traditional custom. They asked us to burn him…They said he had definitely got AIDS” (A 42-year-old woman, in-depth interview.)

The experience of acceptance or exclusion in the community varied a great deal, but frequently the informants in the in depth interviews reported that they were isolated in the community where they lived:

“My neighbours do not approach close to me. They do not want to talk to me. They do not dare to stand next to me. There are a lot of kids in our neighbourhood. Before, the children often came to my house and played. Now, if they come to my house their parents ask them to go home.” (A 22-year-old, man, in-depth interview)

The fear of contagion also spread to limit the social interaction of his or her family members as illustrated by the following quote:

“I disclosed my HIV-positive status to neighbours. People do not want my nephews and nieces to play with their children. People seem to think that HIV is something extremely dangerous. If you touch you will die immediately.” (A 36-year-old man, focus group discussion)

Many told about loss of friends:

“I feel isolated by the society. Before, I had a lot of friends but they have left me over time.” (A 30-year-old man, in-depth interview)

Others withdrew from all social life for fear of being rejected:

“I do not want to socialise any more. I do not want to visit my neighbours any more. I just stay around in my house.” (A 28-year-old man, in-depth interview)

Depression and suicidal thoughts were common:

“I feel very sad and fed up. I do not want to contact anyone. Sometimes, I want to take my own life, but I think about my wife and kid.” (A 32-year-old man, in-depth interview)

But there were also reports about care and support. Many reported being treated normally and even having received a lot of support, especially material support from community members:

“Some of my neighbours are very good and supportive. Some even give me money. The local authority is also supportive. I receive 10kg of rice per month and medicine every 6 months.” (A 30-year-old man, in-depth interview)

#### Care and support from family members

PLHIV received emotional support including affection, comforting, and encouragement from their family members. While the stories about interaction with people outside the family were predominantly framed in a language of ‘stigma and discrimination’, stories about interaction with family members were framed in a language of care and support. The findings indicated family acceptance after the initial shock and fear:

“In the beginning when I learned that I was HIV-positive, both my family and my wife’s family were very scared and shouted at me a lot. But they turned to love and support soon afterward.” (A 32-year-old man, in-depth interview)

Some even expressed that they received more care and support than before, and that this is vital to their spirit and hope for a future, as the following testimony illustrates:

“I think if the spirit goes down, the health will go down as well. My family give all their love to me. They even care about me more than before. They always comfort me to make me optimistic.” (A 35-year-old man, focus group discussion)

As these examples illustrate, the perceptions and experiences of PLHIV of care and support and of stigma and discrimination seemed to vary primarily with emotional proximity and distance. While the Vietnamese society was perceived and experienced as extremely stigmatising and discriminating towards PLHIV, family members were predominantly experienced as supportive and loving after they got over the first shock following disclosure. Parents in particular were tolerant to their HIV-infected daughters or sons.

## Discussion

This study has documented how the double burden of stigma that PLHIV perceive actively prevents them from seeking and utilisation of necessary healthcare and support structures. Experience of discrimination in healthcare settings, at the workplace, and in the local community were commonly reported and appeared to be an important hindrance for PLHIV to seek and utilise HIV-related services.

The high stigma seemed to be rooted in government policies together with misconceptions in the population regarding transmission risk. The government policy based on the “social evil” perception is a case in point here, where the majority of HIV infected in Viet Nam, i.e. the injecting drug users and sex workers, is declared a social evil. Parker and Aggleton conceptualise stigma and discrimination as intimately linked to reproduction of social difference [30]. They argue that stigma feeds upon and reproduces existing inequalities of class, race, gender and sexuality. In this study, the HIV diagnoses came to reinforce the already marginal status of IDU and FSWs. The negative images used by the media and health education posters worked to stimulate and reinforce fear of the disease, nurture negative images of PLHIV, and place the responsibility and the blame on IDUs and FSWs for being the source of the virus and causing its spread. The data forming the basis for this study are from 2007, but the findings remain relevant. In the 2012 Global AIDS Response Progress Report, the government of Viet Nam states that the anti-drug and anti-prostitution policy, stigma and discrimination are major barriers to early HIV diagnosis, access, and adherence to care and treatment services [31].

The documentation that stigma and discrimination are associated with drug addiction and sex work, which are considered social evils, agrees with previous findings in Viet Nam [32-34]. Fear of the disease due to misconceptions about it being a death sentence was stimulated and reinforced by the negative images used by the media and in health education and worked to nurture the negative images of PLHIV. This suggests that efforts to disconnect HIV from the philosophy of social evil, along with proper HIV/AIDS education information provision through public health education programmes, could have a positive effect. The majority of PLHIV in this study were unemployed and has great need of financial support. There is evidence that drug maintenance treatment reduces heroine use, criminal behaviour and injection-related HIV risks [35]. Both harm reduction and comprehensive care and support programmes including employment and financial support are needed to reduce the very high HIV prevalence and criminality among IDUs, and improve the positive image of PLHIV in the general public.

Seeking and utilisation of healthcare services is not only related to distance, transport and cost, but also to the issue of discrimination. This study focused on the narratives of only PLHIV and has not established enacted stigma or discriminatory attitudes among health workers or discriminatory routines in the healthcare system. However, the common experience of being stigmatised and discriminated in healthcare settings accords with previous observations in Viet Nam [20,32,34], as well as in high-income countries, especially among HIV-positive drug users [36,37]. As a result, stigma and discrimination become major barriers to health-seeking behaviour among PLHIV [32,34].

Isolation from the community owing to perceived or enacted stigma and discrimination makes it difficult for care and support services to reach PLHIV. Our previous study reported that fear of rejection and loss of intimacy made disclosure difficult, and this was perceived as a major obstacle to the use of condoms among recently-diagnosed HIV-infected individuals [38].

In Vietnamese culture, it is expected that families stay close to help and support each other during difficult times, and this study showed that the family support was critical. This is consistent with studies in China and Africa that show that the family was the primary source of care and support, especially women [11,39]. Studies in Thailand have indicated that the place in which adult PLHIV commonly pass the advanced stage of disease is their parents’ home, and that the most usual caregivers are parents, particularly the mother, and spouses of married PLHIV [40,41]. Given the impact of HIV on the family and the importance of family in prevention, care and support for PLHIV, HIV becomes a family illness. Therefore, there should be more focus on families with HIV, not just on individuals living with it [42].

The findings indicate the need for improvement of care and support programmes. A home- and community-based model in which care is provided by family members and community care-givers is a possible option. So far, the government has offered no training programmes or financial support to family or community caregivers. Some international non-government organizations have offered financial and technical support for implementing care and support activities, with comprehensive services including medical, psychological, social and financial support for PLHIV. Studies among HIV-positive mothers in Viet Nam suggest that establishing support and self-help groups can diminish the feeling of stigma and facilitate seeking and utilisation of essential and vital services [32,43]. Implementation of the “Greater Involvement of People Living with HIV/AIDS” (GIPA) concept could thus reduce stigma and discrimination, and enable PLHIV to gain seeking and utilisation of care and support.

This study was conducted using the purposeful sampling method amongst only 45 men and women who were living with HIV in a well-established concentrated HIV epidemic and in which the responses to the HIV epidemic is quite comprehensive, including HIV/AIDS prevention. Moreover, Hai Phong is a very urban city, while many of the remaining 62 cities/provinces in the country are non-urban, mountainous, with isolated and limited investments in HIV/AIDS response. Hence, the findings from this study might not be transferable to all areas of the country. Furthermore, other community members, such as health care providers and family members of PLHIV, were not included, so the sources of information were limited. However, given the continuation of national anti-drug and anti-prostitution policy, we argue that the findings and recommendations are relevant in areas with high prevalence of HIV and injecting drug use.

## Conclusions

Stigma and discrimination continue to be major obstacles to seeking and utilising HIV essential services for PLHIV in Viet Nam. The findings underscore the importance of family members in care and support; they should be given central roles in HIV programming. A comprehensive care and support programme along with proper HIV/AIDS education information provision needs to be carefully developed. Drastic policy changes addressing the ‘social evil’ perception of IDUs, FSWs, and PLHIV are needed to improve the utilisation of HIV/AIDS essential services and to make HIV/AIDS intervention efforts effective.

## Competing interests

The authors declare that they have no competing interests.

## Authors’ contributions

All authors contributed to the paper; all authors conceived the study. DCT conducted the study. All authors helped to conceptualise ideas and interpret the findings. DCT prepared the drafts, and the other authors reviewed and finalised the manuscript.

## Pre-publication history

The pre-publication history for this paper can be accessed here:

http://www.biomedcentral.com/1472-6963/12/428/prepub

## References

[B1] Green G Social support and HIV AIDS Care 1993 5 1 87 104 10.1080/09540129308258587 8461365

[B2] Mizuno Y Purcell DW Dawson-Rose C Parsons JT Correlates of depressive symptoms among HIV-positive injection drug users: the role of social support AIDS Care 2003 15 5 689 698 10.1080/09540120310001595177 12959820

[B3] Chesney MA Chambers DB Taylor JM Johnson LM Social support, distress, and well-being in older men living with HIV infection J Acquir Immune Defic Syndr 2003 33 Suppl 2 S185 S193 12853868 10.1097/00126334-200306012-00016

[B4] Kimberly JA Serovich JM The role of family and friend social support in reducing risk behaviors among HIV-positive Gay men AIDS Educ Prev 1999 11 6 465 475 10693644

[B5] Reilly T Woo G Social support and maintenance of safer sex practices among people living with HIV/AIDS Health Soc Work 2004 29 2 97 105 10.1093/hsw/29.2.97 15156842

[B6] Colindres R Mermin J Ezati E Kambabazi S Buyungo P Sekabembe L Baryarama F Kitabire F Mukasa S Kizito F Utilization of a basic care and prevention package by HIV-infected persons in Uganda AIDS Care 2008 20 2 139 145 10.1080/09540120701506804 17896196

[B7] Kalichman SC DiMarco M Austin J Luke W DiFonzo K Stress, social support, and HIV-status disclosure to family and friends among HIV-positive men and women J Behav Med 2003 26 4 315 332 10.1023/A:1024252926930 12921006

[B8] Ncama BP Acceptance and disclosure of HIV status through an integrated community/home-based care program in South Africa Int Nurs Rev 2007 54 4 391 397 10.1111/j.1466-7657.2007.00560.x 17958669

[B9] Knowlton AR Hua W Latkin C Social support networks and medical service use among HIV-positive injection drug users: implications to intervention AIDS Care 2005 17 4 479 492 10.1080/0954012051233131314349 16036234

[B10] MacNeil JM Mberesero F Kilonzo G Is care and support associated with preventive behaviour among people with HIV? AIDS Care 1999 11 5 537 546 10.1080/09540129947695 10755029

[B11] Li L Wu S Wu Z Sun S Cui H Jia M Understanding family support for people living with HIV/AIDS in Yunnan, China AIDS Behav 2006 10 5 509 517 10.1007/s10461-006-9071-0 16741672 PMC2795327

[B12] Kalichman SC Sikkema KJ Somlai A People living with HIV infection who attend and do not attend support groups: a pilot study of needs, characteristics and experiences AIDS Care 1996 8 5 589 599 10.1080/09540129650125542 8893909

[B13] Mahajan AP Sayles JN Patel VA Remien RH Sawires SR Ortiz DJ Szekeres G Coates TJ Stigma in the HIV/AIDS epidemic: a review of the literature and recommendations for the way forward AIDS 2008 22 Suppl 2 S67 S79 10.1097/01.aids.0000327439.20914.33 18641472 PMC2835402

[B14] UNAIDS UNAIDS fact sheet on stigma and discrimination 2003 Geneva http://data.unaids.org/publications/Fact-Sheets03/fs_stigma_discrimination_en.pdf

[B15] Scambler G Stigma and disease: changing paradigms Lancet 1998 352 9133 1054 1055 10.1016/S0140-6736(98)08068-4 9759769

[B16] UNAIDS HIV - Related Stigma, Discrimination and Human Rights Violations - Case studies of successful programmes UNAIDS Best practice collection 2005 Geneva 7 10 http://data.unaids.org/publications/irc-pub06/jc999-humrightsviol_en.pdf

[B17] Wolitski RJ Pals SL Kidder DP Courtenay-Quirk C Holtgrave DR The Effects of HIV Stigma on Health, Disclosure of HIV Status, and Risk Behavior of Homeless and Unstably Housed Persons Living with HIV AIDS Behav 2008 13 6 1222 1232 18770023 10.1007/s10461-008-9455-4

[B18] Kaplan AH Scheyett A Golin CE HIV and stigma: analysis and research program Curr HIV/AIDS Rep 2005 2 4 184 188 10.1007/s11904-005-0014-6 16343376

[B19] Gaudine A Gien L Thuan TT Dung do V: Perspectives of HIV-related stigma in a community in Vietnam: a qualitative study Int J Nurs Stud 2010 47 1 38 48 10.1016/j.ijnurstu.2009.06.004 19729162

[B20] Thi MD Brickley DB Vinh DT Colby DJ Sohn AH Trung NQ le Giang T Mandel JS A qualitative study of stigma and discrimination against people living with HIV in Ho Chi Minh City, Vietnam AIDS Behav 2008 12 4 Suppl S63 S70 18360743 10.1007/s10461-008-9374-4

[B21] Nguyen TH Nguyen TL Trinh QH HIV/AIDS epidemics in Vietnam: evolution and responses AIDS Educ Prev 2004 16 3 Suppl A 137 154 15262572 10.1521/aeap.16.3.5.137.35527

[B22] MoH Results from the HIV/STI Integrated Biological and Behavioral Surveillance (IBBS) in Vietnam 2007 Vietnam Administration for AIDS Control (VAAC), Ministry of Health (MoH), Ha Noi, Viet Nam

[B23] Khuat TH HIV/AIDS policy in Viet Nam: A Civil Society Perspective 2007 http://www.soros.org/initiatives/health/focus/phw/articles_publications/publications/VietNam_20071129

[B24] MoH The third country report on following up the implementation to the declaration of commitment on HIV and AIDS Vietnam Administration for AIDS Control (VAAC), Ministry of Health (MoH) 2008 Ha Noi, Viet Nam

[B25] MOH National HIV/AIDS Annual Report 2008 Vietnam Administration for AIDS Control (VAAC), Ministry of Health (MoH), Ha Noi, Viet Nam

[B26] NIHE Survey on the realities of care, counselling, support to HIV/AIDS infected cases and community-based HIV interventions in Viet Nam. National Institute of Hygiene and Epidemiology (NIHE) 2005 Ha Noi, Viet Nam

[B27] Thanh DC Hien NT Tuan NA Thang BD Long NT Fylkesnes K HIV risk behaviours and determinants among people living with HIV/AIDS in Vietnam AIDS Behav 2009 13 6 1151 1159 10.1007/s10461-008-9451-8 18787940

[B28] Hammett TM Wu Z Duc TT Stephens D Sullivan S Liu W Chen Y Ngu D Des Jarlais DC ‘Social evils’ and harm reduction: the evolving policy environment for human immunodeficiency virus prevention among injection drug users in China and Vietnam Addiction 2008 103 1 137 145 10.1111/j.1360-0443.2007.02053.x 18028519

[B29] DOH Provincial HIV/AIDS annual report 2006 Hai Phong Department of Health (DOH), Hai Phong, Viet Nam

[B30] Parker R Aggleton P HIV and AIDS-related stigma and discrimination: a conceptual framework and implications for action Soc Sci Med 2003 57 1 13 24 10.1016/S0277-9536(02)00304-0 12753813

[B31] GOV The 2012 Global AIDS Response Progress Report 2012 Goverment of Viet Nam (GOV), Ha Noi, Viet Nam

[B32] Nguyen TA Oosterhoff P Ngoc YP Wright P Hardon A Self-help groups can improve utilization of postnatal care by HIV-infected mothers J Assoc Nurses AIDS Care 2009 20 2 141 152 10.1016/j.jana.2008.10.006 19286126

[B33] Khuat TH Nguyen TVA Og J Understanding HIV and AIDS-related stigma and discrimination in Vietnam Institute for Social Development Studies and International Center for Research on Women 2004 Ha Noi, Viet Nam

[B34] Maher L Coupland H Musson R Scaling up HIV treatment, care and support for injecting drug users in Vietnam Int J Drug Policy 2007 18 4 296 305 10.1016/j.drugpo.2006.12.006 17689378

[B35] Institute of Medicine Preventing HIV Infection Among Injecting Drug Users in High-Risk Countries: An Assessment of the Evidence 2006 Washington, DC, USA: National Academies Press http://www.iom.edu/~/media/Files/Report%20Files/2006/Preventing-HIV-Infection-among-Injecting-Drug-Users-in-High-Risk-Countries-An-Assessment-of-the-Evidence/11731_brief.ashx 10.1080/09540120903193757 26876272

[B36] Surlis S Hyde A HIV-positive patients’ experiences of stigma during hospitalization J Assoc Nurses AIDS Care 2001 12 6 68 77 10.1016/S1055-3290(06)60185-4 11723915

[B37] Elford J Ibrahim F Bukutu C Anderson J HIV-related discrimination reported by people living with HIV in London, UK AIDS Behav 2008 12 2 255 264 10.1007/s10461-007-9344-2 18080829

[B38] Thanh DC Moland KM Fylkesnes K The context of HIV risk behaviours among HIV-positive injection drug users in Viet Nam: Moving toward effective harm reduction BMC Public Health 2009 9 1 98 10.1186/1471-2458-9-98 19348681 PMC2676271

[B39] Kipp W Tindyebwa D Rubaale T Karamagi E Bajenja E Family caregivers in rural Uganda: the hidden reality Healthcare Women Int 2007 28 10 856 871 10.1080/07399330701615275 17987457

[B40] Vithayachockitikhun N Family caregiving of persons living with HIV/AIDS in Thailand: caregiver burden, an outcome measure Int J Nurs Pract 2006 12 3 123 128 10.1111/j.1440-172X.2006.00560.x 16674778

[B41] Knodel J VanLandingham M Saengtienchai C Im-em W Older people and AIDS: quantitative evidence of the impact in Thailand Soc Sci Med 2001 52 9 1313 1327 10.1016/S0277-9536(00)00233-1 11286358

[B42] Rotheram-Borus MJ Flannery D Rice E Lester P Families living with HIV AIDS Care 2005 17 8 978 987 10.1080/09540120500101690 16176894

[B43] Oosterhoff P Anh NT Yen PN Wright P Hardon A HIV-positive mothers in Viet Nam: using their status to build support groups and access essential services Reprod Health Matters 2008 16 32 162 170 10.1016/S0968-8080(08)32408-2 19027632

